# Absence of Exceptional Points in Square Waveguide Arrays with Apparently Balanced Gain and Loss

**DOI:** 10.1038/srep22711

**Published:** 2016-03-07

**Authors:** Zhenzhen Liu, Qiang Zhang, Xiangli Liu, Y. Yao, Jun-Jun Xiao

**Affiliations:** 1 College of Electronic and Information Engineering, Shenzhen Graduate School, Harbin Institute of Technology, Xili, Shenzhen 518055, China; 2 Department of Materials Science and Engineering, Shenzhen Graduate School, Harbin Institute of Technology, Xili, Shenzhen 518055, China

## Abstract

The concept of parity-time (PT) symmetry in the field of optics has been intensively explored. This study shows the absence of exceptional points in a three-dimensional system composed of a square waveguide array with diagonally-balanced gain/loss distribution. More specifically, we show that an array of four coupled waveguides supports eight fundamental propagation supermodes, four of which are singlet, and the other two pairs are double degenerated. It is found that the singlet states follow the routine PT phase transition; however, the double-degenerated modes never coalesce as the gain/loss-to-coupling strength level varies, showing no actual PT symmetry-derived behavior. This is evident in the phase rigidity which does not approach zero. The absence of exceptional points is ascribed to the coupling of non-symmetric supermodes formed in the diagonal waveguide pairs. Our results suggest comprehensive interplay between the mode pattern symmetry, the lattice symmetry, and the PT-symmetry, which should be carefully considered in PT-phenomena design in waveguide arrays.

In the realm of quantum mechanics, the spectrum of a Hermitian Hamiltonian is ensured to be completely real and positive^1^. However, it is only a sufficient condition, not a necessary one. For a non-Hermitian Hamiltonian, the spectrum can also be real in the cases in which the Hamiltonian is parity-time (PT) symmetric (i.e., *PTH* = *HPT*)^2^,^3^,^4^,^5^, belonging to a more general class of pseudo-Hermitian systems. The intriguing properties are mainly related to the exceptional points (EPs)^6^,^7^ which are the crossing points of eigenvalues trajectories. At these points, two corresponding eigenfunctions are linearly dependent and their eigenvalues coincide. In approaching the EPs, a dynamical phase transition takes place. Systems with PT-symmetry have recently been a topic of interest in several frontiers in physics, including quantum field theories^8^, non-Hermitian Anderson models^9^, and open quantum systems^5^,^10^, to name a few.

Furthermore, the equivalence of the Schrödinger equation in quantum mechanics with the paraxial wave equation of the approximated Maxwell’s equations^11^, leads to the application of the concept of PT symmetry toward many optical systems. To date, there have been numerous fascinating phenomena reported in optical structures bearing PT symmetry: single-mode lasers^12^, unidirectional light propagation^13^, coherent absorption^14^, micro-ring and micro-disk resonators^15^, whispering-gallery cavities^16^ and others^17^,^18^,^19^,^20^, to mention a few. In optics, the complex refractive index profile plays the role of complex potential, so intuitively the system should be PT symmetric if it satisfies *n*(*x*) = n^*^(−*x*) (* stands for complex conjugate), i.e., the real part and imaginary part of the index are of even and odd function, respectively. However, more significantly important is that the realization of PT is generally associated to the coupling and hybridized modes, abbreviated as “H-mode” in nanophotonic systems. It is these H-modes whose modal index should meet the requirement 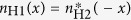, where H1 and H2 represent the elementary modes that couple to each other and form a supermode over the whole system, that really matters in terms of PT-symmetry breaking. Apparently one way to realize the requirement of modal index is to introduce balanced gain/loss 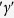 in appropriately selected positions. Generally, gain or loss can be achieved through quantum well, erbium doping^16^ or photorefractive structures^17^ at the conduction band. Interestingly, when the factor *γ* increases (i.e., by imposing more gain/loss), there an EP appears at which the system switches from PT-unbroken phase (both modes with real propagation constant) to broken PT-symmetric phase (with complex conjugate eigenvalues) where one mode is amplified while the other is attenuated. It is noticed that even for asymmetric directional couplers, the system also exhibits behavior resembling that of PT symmetric systems^21^.

There are many studies about 2D and quasi-2D waveguide PT-symmetric systems^22^,^23^. In these structures, the modal effective index is determined by the material index where the guiding mode is concentrated and highly localized. In a previous work, the absence of EP has been observed in a finite 1D waveguide array^24^. However, the mechanism is different to ours reported here. Three central waveguides are depleted off the gain/loss so that particular supermodes maintain exactly real eigenvalues. While for the 3D system of four closely coupled waveguides we study here, the system loses the EP for particular supermodes that have accidentally non-equal real part of the eigenvalue. The evolution of the eigen-index trajectories in such a non-Hermitian Hamiltonian system is controlled by the external parameter (e.g. gain/loss amount *γ*) and more importantly depends on the modal symmetry and the lattice symmetry. The system show different coupling interactions and distinct properties as compared to the 1D system^24^. Combining the spatial coupled mode theory (SCMT)^21^,^25^,^26^ and finite element method (FEM)^27^, we analytically and numerically depict the process and the underlying mechanism, particularly for the absence of the EP that emerges for four particular bands.

## Results

### PT-symmetry induced mode splitting in two coupled waveguides with and without gain/loss

**Figure 1:**
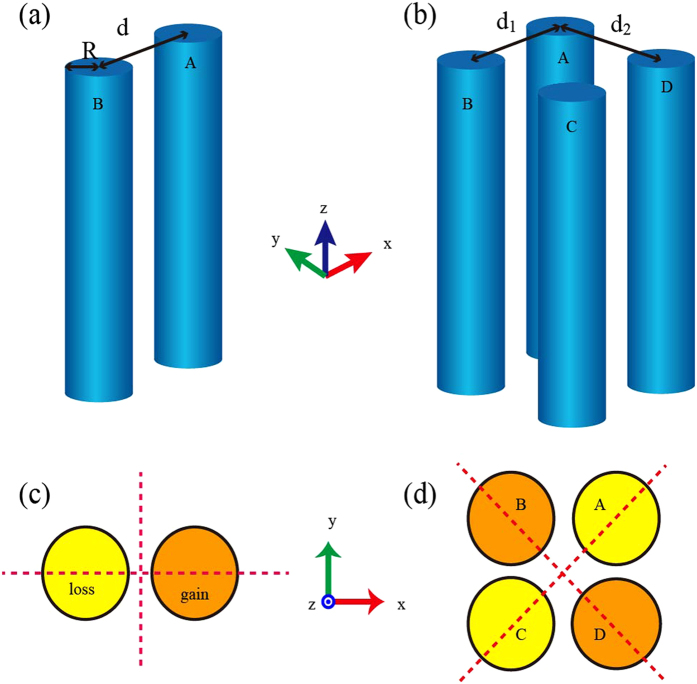
Schematics of the proposed 3D coupled waveguides and cross section profile. Schematic figures of the coupled waveguides system composed of double waveguides (**a**) and four waveguides (**d**), respectively. (**c**) and (**d**) represent the cross sectional view corresponding to (**a**) and (**b**) with the following dimensions: *R* = 0.2 μm and 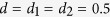 μm. In this configuration, the structure has a high geometrical symmetry.

**Figure 2:**
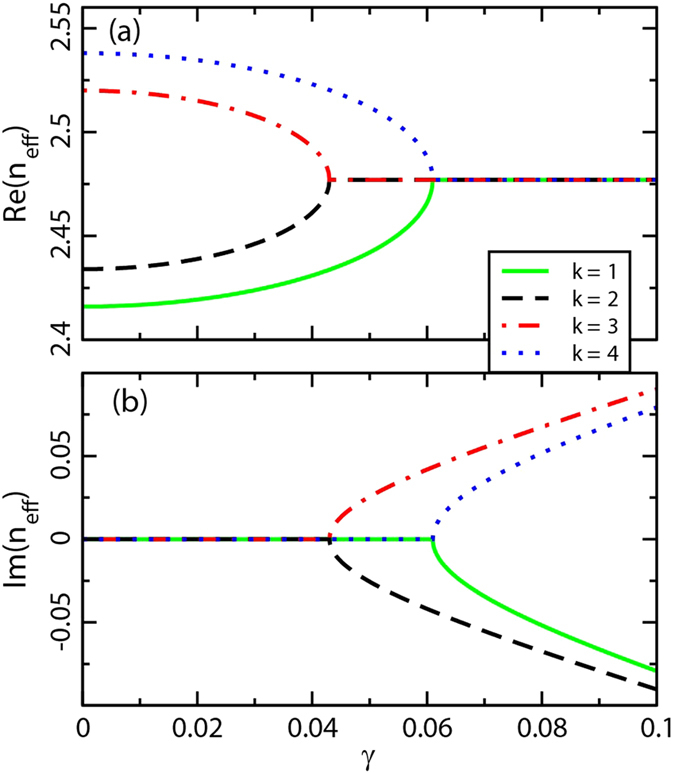
Evolution of eigen-indices with increasing unperturbed gain/loss factor *γ*. (**a**) Real and (**b**) imaginary parts of four supermodes *n*_*eff*_ for a Hamiltonian *H* as a function of *γ*. The parameters used in the theoretical analysis are *n*_*e*_ = 2.477, *κ*_*T*_ = 0.043, and *κ*_*L*_ = 0.061.

To describe the PT behaviors of the four-waveguide system, it is instrumental to fully understand the PT symmetry associated properties of the elementary cell, i.e., the double coupling waveguides. Figure 1a schematically shows the coupled double-waveguide system with two identical cylinder waveguides. We note that the unperturbed propagation constant is *β* = *n*_*e*_*k*_0_ (i.e., the case for no coupling and without gain/loss), where *n*_*e*_ is the effective fundamental modal index, *k*_0_ = 2*π*/*λ*_0_, and *λ*_0_ is the vacuum wavelength^28^. For the configuration that gain is set in cylinder A and equal amount of loss is assumed in cylinder B, the system can be mathematically represented by the SCMT (see Methods):


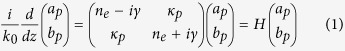


where 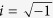 and *a*_*p*_ and *b*_*p*_ (*p* = *T,L*) are the amplitudes of the unperturbed propagation modes inside the individual waveguide A and B, respectively. In Eq. (1), *γ* is the effective gain/loss quantity based on the imaginary part of the refractive index, and *κ*_*L*_ (*κ*_*T*_) denotes the coupling strength for the longitudinal (transverse) case. It is noted that the terminologies ‘transverse’ and ‘longitudinal’ here refer to the modal coupling type at the cross section. This is different to the commonly used ‘longitudinal mode’ for bulk plasmon^29^ or acoustic system^30^. The eigenvalues of the Hamiltonian *H* are:


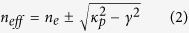


with +(−) corresponding to bonding (anti-bonding) hybrid mode. When γ increases, 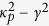 becomes negative at a particular point, inevitably leading to a broken PT-symmetric phase with complex conjugate eigenvalues. The real and imaginary parts of Eq. (2) are shown in Fig. 2a,b, respectively, in which *n*_*e*_ = 2.477, *κ*_*T*_ = 0.043 and *κ*_*L*_ = 0.061. Figure 2 represents a good example by which to clarify the EP behavior; it clearly shows a transition from the PT-symmetric phase to the broken PT-symmetric phase at *γ* = 0.043 (for longitudinal case) and *γ* = 0.061 (for transverse case), respectively.

**Figure 3:**
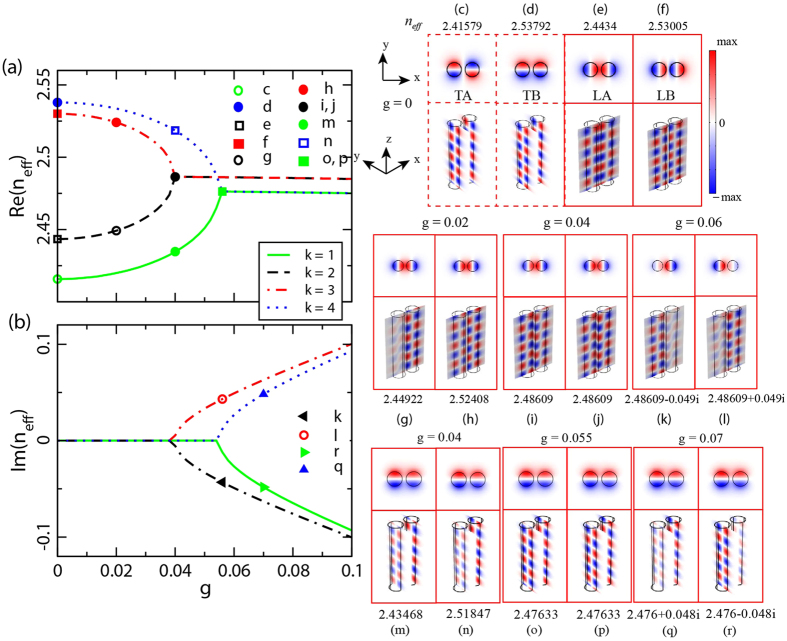
Numerical results of the evolution of eigen-indices with increasing unperturbed gain/loss factor *g* for double coupled waveguides. (**a**) Real and (**b**) imaginary parts of four supermodes *n*_*eff*_ (Fig. 1(a)) as a function of *g*, obtained by FEM numerical simulation. For each mode without gain/loss injection, i.e., *g* = 0, the field distribution *E*_*z*_ in the transverse section and longitude surface are shown in (**c**–**f**). For the group of (**e**,**f**), as g is increasing from the unbroken to the broken phase, the field distributions are in accordance with (**g**,**h**), (**i**,**j**) and (**k**,**l**), respectively. (**m**–**r**) transverse modes corresponding to (**c**,**d**).

In addition to the above theoretical analysis, it is possible to numerically demonstrate this PT-symmetry induced phase transition in a real system (see Methods). In this study, we set the wavelength at *λ*_0_ = 1.55 μm. The two cylinders (A,B in Fig. 1c) have radii *R* = 0.2 μm with a gain/loss factor *g*, i.e. the imaginary part of the waveguide refractive index. The two waveguides are at center-to-center separation *d* = 0.5 μm. The various refractive indices associated with the structure are shown in Table 1. In this case, approximately *γ* (the imaginary part of the effective index) is lineally related to the factor *g* as *γ* ≈ 1.129 *g*. This relationship is obtained by numerical simulation for the isolated waveguide (Fig. S2 in Supplementary Information). Figures 3a,b show the numerical results of the coupling effects, by plotting the dependence of the supermode refractive index on gain/loss amount *g*. It is seen in Fig. 3a that there are a total of four bands (labeled consecutively by *k* = 1, 2, 3, 4), and there is a small shift of Re(*n*_*eff*_) [refer to 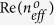 in Table 1] for the longitudinal (black and red) and the transverse case (blue and green) in the broken PT-symmetric phase, as expected. We note that to illustrate this mode coupling symmetry induced effect, a general coupled mode theory has been developed for a similar non-Hermitian system^31^. The real part of *z*-component of the electric field at the waveguide cross section and at a slice along the propagation direction (+*z*) are shown in the right panels of Fig. 3, for points correspondingly labeled in Fig. 3a,b. More specifically, Fig. 3c–f show the cases of the unperturbed modes when *g* = 0 (Hermitian system), corresponding to the cases of transverse anti-bonding (TA), transverse bonding (TB), longitudinal anti-bonding (LA), and longitudinal bonding (LB), respectively. We stress that all of the four modes will be treated as the fundamental mode elements for further discussion in the four coupled waveguides. The effective index of these coupled modes is 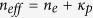 (for bonding modes) and 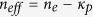 (for anti-bonding modes) according to Eq. (2), where *p* = *L,T* for the longitudinal and transverse cases, respectively. As the gain/loss factor *g* increases, the modes for bands *k* = 1, 4 and *k* = 2, 3 cease to be orthogonal; they become mixed. More specifically, for bands *k* = 2, 3, the coupling effect is large enough to compensate the loss before the EP, as shown in Fig. 3g,h where *g* = 0.02. For systems close to the EP, the balanced coupling strength and gain/loss yield two propagation modes that are indistinguishable (fully mixing), as shown in Fig. 3i,j. Once the gain/loss exceeds the EP, one of the double bands (*k* = 2) changes its state to ‘loss’, and the other (*k* = 3) changes its state to ‘gain’, accompanied by the energy concentration inside the loss waveguide B and gain waveguide A, respectively. It is easy to recognize this by the variation in field amplitude (Fig. 3k,l) in both the waveguide cross section and the propagation direction. Similar behaviors for the transverse modes are shown in Fig. 3m–r.

### PT-symmetry induced properties of waveguide array in a square lattice

**Figure 4:**
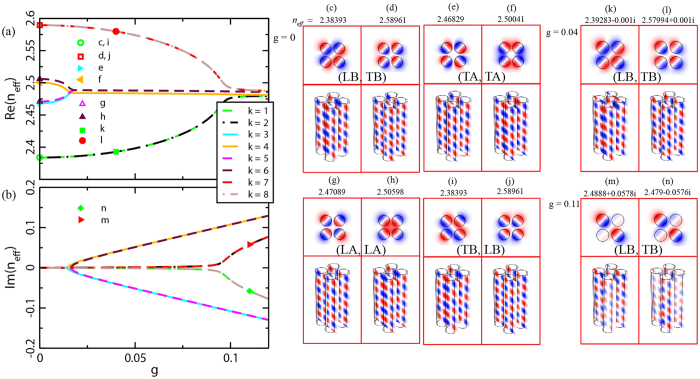
Numerical results of the evolution of eigen-indices with increasing unperturbed gain/loss factor *g* for the four coupled waveguides. (**a**) Real and (**b**) imaginary parts of total eight supermodes *n*_*eff*_ (Fig. 1b) as a function of *g*, obtained by FEM numerical simulation. For each mode without gain/loss injection, i.e. *g* = 0, the field distribution *E*_*z*_ in the transverse section and longitude surface are shown in (**c**–**j**) corresponding to eight line shapes respectively. For the group of (**c**,**d**), as *g* is increasing, the field distributions are shown in (**k**,**l**) and (**m**,**n**), respectively.

From the viewpoint that both longitudinal and transverse cases in the double waveguides situation exist, it is interesting to explore the situation of four coupled waveguides (Fig. 1b) which can be regarded as a system consisting of two coupled double-waveguides. Figure 1d depicts the geometry in a square lattice (*d*_1_ = *d*_2_ = 0.5 μm) and has balanced gain/loss in the diagonal waveguides. For the sake of a simplified discussion, let us divide the system into two subunits: one containing waveguides A and C (labeled as AC) and the other containing B and D (labeled as BD). Figure 4 shows the numerically calculated effective index of the eight propagation supermodes labeled as 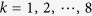 respectively, for various gain/loss amounts *g*. It is seen that four of the bands, i.e. *k* = 3, 4 and *k* = 5, 6 in Fig. 4a,b, have clear PT symmetry associated characteristics similar to the double-waveguides system. However, there are two groups of double-degenerated modes, i.e., *k* = 1, 7 and *k* = 2, 8 in Fig. 4a,b, that show unexpectedly dissimilar properties. Specifically, as the gain/loss amount increases, apparently no EP shows up and the upper and lower bands do not really coalesce. The corresponding *E*_*z*_ pattern of the eigenstates in absence of gain/loss (*g* = 0) are shown in Fig. 4c–j, respectively. We note that any of the supermodes can be considered as the coupling results of the sub-modes formed in the diagonal double-waveguides (e.g., AC and BD). With regard to the high lattice symmetry and mode orthogonality, there are four coupling situations emerging in the system, respectively, in bonding or anti-bonding fashion. The counterintuitive phenomenon occurs in the LB and TB coupling case which does not possess absolute EP (for bands *k* = 1, 7 and *k* = 2, 8), even though apparently the structure meets the condition *n*(*x*) = n^*^(−*x*). The modes in bands *k* = 1, 7 for *g* = 0.04 and *g* = 0.11 are shown in Fig. 4k,l and Fig. 4m,n, respectively. Although one of the modes is a ‘gain’ state (Fig. 4m) and the other is a ‘loss’ state (Fig. 4n) for *g* = 0.11, the real part of the refractive index are not equal, which is not as expected. This forbids the transition from the PT symmetric phase to the broken PT symmetric phase. Despite of the similar mode profiles of Re(*E*_*z*_) [top panels in Fig. 4m,n] at a cross section, the evolutions of the energy distributions [bottom panels in Fig. 4m,n] are quite distinguishing between the gain and loss states as the waves propagate along the waveguides. To illustrate the differences more clearly, we specifically select the two groups composed of bands *k* = 1, 7 and bands *k* = 3, 4 in the following discussion.

**Figure 5:**
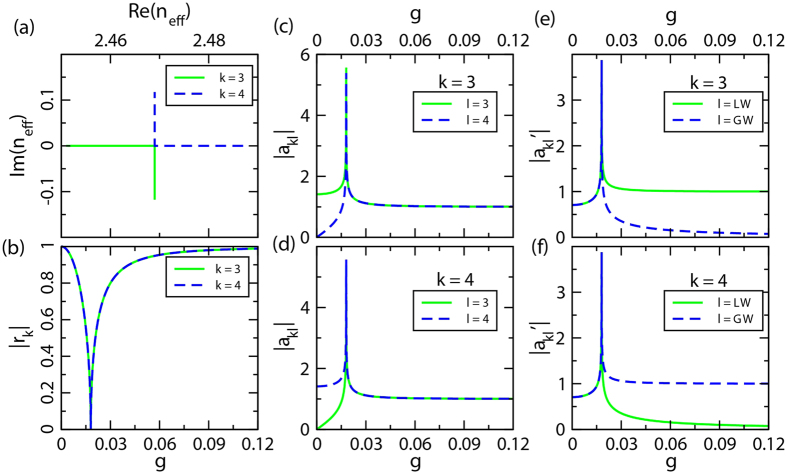
The description of eigenstates *k* = 3, 4 as a function of *g* through variant patterns to characterize different properties. (**a**) Trajectory of eigen-indices *n*_*eff*_ in the complex frequency plane. (**b**) Norm of phase rigidity 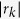 for each of the chosen states. (**c**,**d**) Evolution of eigenstate-*k* = 3, 4 as a function of gain/loss *g* in the mode representation, i.e., projecting each eigenstate onto the double original state simultaneously, in which 
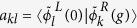
, and 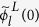 (*l* = 3, 4) are eigenstates at *g* = 0. (**e**,**f**) Similar to (**c**,**d**), they are the evolution in the gain/loss waveguides representation, 
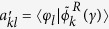
, where 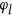 (*l* = LW,GW) are unit basis vectors. Fitted parameters used here are *n*_*e*_ = 2.477, *κ*_*T*_ = 0.008, K_1_ = 0.018.

For the bands *k* = 3, 4 which comply with a PT symmetric case, the double hybrid modes (cf., Fig. 4e,f) are the results of modes coupling between TA (formed in waveguides BD) and TA (formed in waveguides AC) with respectively modal indices 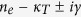. Their coupling strength is denoted by K_1_ and the total system can be described by a determined Hamiltonian:


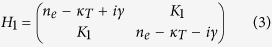


with eigenvalues 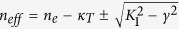. Figure 5a shows an anti-crossing line-shape with a crossing point in the complex plane of effective index. For the Hamiltonian *H*_1_, the phase rigidity^5^  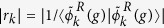 (see Methods) for each state in bands *k* = 3, 4 as a function of *g* is shown in Fig. 5b. It is evident that the phase rigidity 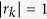 when *g* = 0, indicating absolute purity of the two states. Meanwhile the phase rigidity 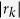 approaches zero at EP, which implies a complete coalescence of the two states. Note that 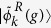 is the normalized right eigenvector under the parameter (*g*) variation. Once 
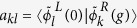
 is obtained, as shown in Fig. 5c,d, we can evaluate the mixing degree of the double states. Although they are equal after the EP, this does not mean that the double states become completely identical. To illustrate that, we introduce another measurement 
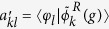
. Here, 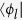 (*l* = 3, 4) is the unit basis vector, corresponding to the gain (GW) and loss (LW) waveguides representation, respectively. The results are shown in Fig. 5e,f for bands *k* = 3, 4. It is seen that beyond the EP, the state with the negative imaginary part is more concentrated in the loss waveguides AC, while the other state, which is of positive imaginary refractive index, is more concentrated in the gain waveguides BD. The properties of the PT symmetric phase transition are demonstrated clearly by such analysis.

Next, we proceed to discuss the bands *k* = 1, 7 which represent non-PT-symmetric cases. Here, the modes LB (formed in the waveguides BD) and TB (formed in the waveguides AC) are coupled to form a pair of supermodes (cf., Fig. 4c,d). However, their modal indices are no longer complex conjugated. The system can now be described by a Hamiltonian:


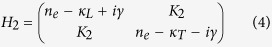


**Figure 6:**
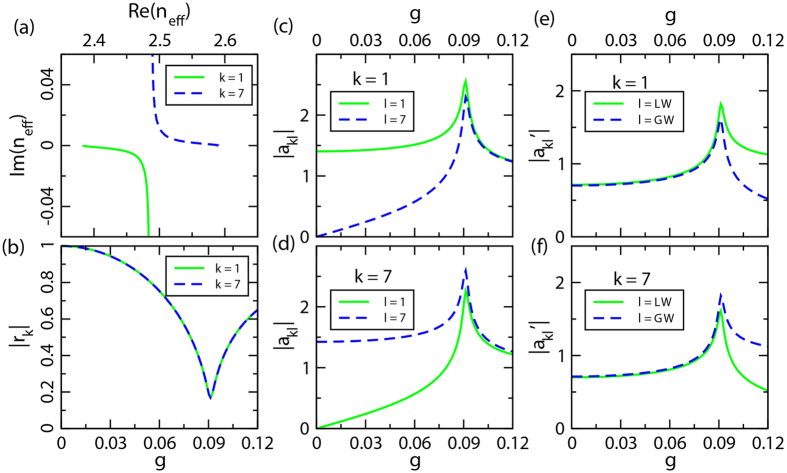
Similar to Fig. 5 with the eigenstates *k* = 1, 7. Fitted parameters used here are *n*_*e*_ = 2.477, 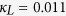, 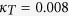, 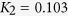.

Note that 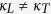 and the LB and TB modes have the coupling strength K_2_. The eigenvalues of *H*_2_ are 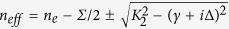, here Δ = *κ*_*L*_ − *κ*_*T*_ and ∑ = *κ*_*L*_ + *κ*_*T*_. In this regard, no real singularity exists for the square root 
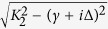
. Similar to the bands *k* = 3, 4, we also calculate the phase rigidity *r*_*k*_ and the evolution of eigenstates as a function of *g* in different types (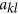 and 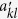 with *k* = 1, 7 and *l* = LW,GW). The results are shown in Fig. 6 which is in sharp contrast to Fig. 5. Figure 6a clearly shows that the real part and imaginary part of the eigenstates become anti-crossing, without a converging point. In addition, their phase rigidity no longer descends to zero, but instead it turns back at 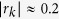 for both bands *k* = 1, 7 (Fig. 6b). This strongly suggests that the double states never become identical. Furthermore, 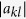 (Fig. 6c,d) are approximately equal for the double states after a certain point, but a small difference between them always exists. Also, for the spectrum of 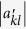 (Fig. 6e,f), the main difference with respect to the PT symmetric case (Fig. 5e,f) is that they are never completely equal. All of these observations are ascribed to the mode pattern with different symmetry. As a matter of fact, the state of *k* = 1 is more concentrated in the waveguides AC (with TB mode) where loss is set, while the state of *k* = 7 is more concentrated in the waveguides BD (with LB mode) where gain is in presence. It is therefore conclusive that the bands *k* = 1, 7 lose their absolute EP; these states can no longer be identical. The exemplificative field distribution of bands *k* = 1, 7 as shown in Fig. 4k–n are consistent with the results shown in Fig. 6.

In view of the supermodes features (see Fig. 4), we study the propagation behavior by selectively exciting the gain or loss waveguides (see Fig. S3 in Supplementary Information). For relatively small parameter *g* = 0.05 there is a clear energy transfer between the gain and loss waveguides over certain propagation length. However, the energy is more confined in the gain waveguides after a sufficient long propagation distance, due to the absence of EP (in which case the supermodes always have complex conjugated propagation constant and the imaginary parts are opposite to each other). While for relatively larger *g* = 0.11, the energy basically concentrates on the gain waveguides, quite similar to the typical PT-symmetric behavior^11^. However, the phase difference of the field in the waveguides is no longer *π*/2 which is one of the characteristic of the PT-symmetry broken phase for states beyond the EP^11^.

To this end, the absence of EP in square waveguide arrays has been verified both in numerical simulation and by theoretical analysis. We stress that the PT-associated phase transition always exists in the waveguide array if the balanced gain/loss is in the orthogonal direction (Fig. S4 in Supplementary Information), or if the waveguide array is tuned from square lattice to rectangular lattice where 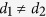 (Fig. S5 in Supplementary Information). In these cases, the lattice symmetry does not match with the modal symmetry.

## Discussion

In summary, a few comments are in order. Firstly, the mode-coupling properties in 3D waveguide arrays with balanced gain/loss distribution have been theoretically and numerically studied. We mainly investigated the phase transition from the unbroken to the broken region across the so-called EP. In a square waveguide array with diagonally balanced gain and loss, as a counterintuitive phenomenon, EP is absent in a particular configuration which has a high lattice symmetry. The effect originates from the mode coupling with different distribution symmetry, which yields an unequal real part of the effective modal index. Secondly, this example strongly demonstrates that an optical system with complex potential meeting the condition 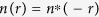 cannot guarantee the PT symmetric transition. It is the modal index of the supermode that actually matters. In this sense, the geometrically symmetric structure with balanced gain/loss is only one of the prerequisites for the emergence of PT symmetric to broken PT symmetric phase transition. Lastly, for experimental demonstration, the waveguides system may be fabricated by direct laser writing technology^32^,^33^, while control over gain and loss of individual waveguides could be achieved through spatial modulation of the optical pump intensity^11^. To observe the consequences of EP and the absence of EP, the input signal should be selectively launched via the gain (or loss) waveguides, then the information of field intensity and the phase difference between the waveguides can be mapped, similar to ref. [11].

## Methods

### Phase rigidity

The phase rigidity is defined as^5^,^34^:


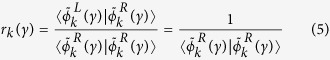


where 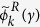 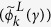 is the normalized right (left) eigenvector of the Hamiltonian matrix for band *k* based on the bilinear product of the non-Hermitian Hamiltonian. Phase rigidity is a measure of the mixing degree of different eigenstates. The phase rigidity varies between 1 in the regime of well isolated resonance and 0 in the regime of overlapping resonances (i.e., crossing point). For zero *γ* (i.e., a Hermitian system), the eigenstates are distinct and orthogonal, their phase rigidity is close to unity. As *γ* increases, the states become highly mixed. At the EP, the phase rigidity vanishes because of the completely coalesced states. Another way to characterize an EP is to trace the evolution of the eigenstates (i.e., 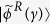) under a parameter variation. By projecting each eigenvector of band *k* onto their original states, i.e., when *γ* = 0, represented by 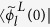), 
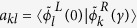
 it is proposed to quantitatively denote the *k* proportion of each band of the respective original states. By substituting the unit basis vector 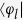 for the original state 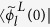, the operator 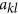 becomes 
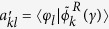
, which is used to determine where modes of band *k* is most concentrated in the waveguide array.

### Hamiltonian of both longitudinal and transverse couplings

For a cylinder waveguide, it supports double-degenerated fundamental modes which are orthogonal. When it is coupled to another waveguide, there are two independent coupling cases: one for transverse coupling with coupling strength *κ*_*T*_ and the other for longitudinal coupling with corresponding coupling strength *κ*_*L*_, which can be obtained by spatial coupled mode theory:


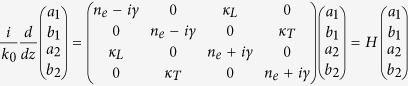


It is seen that this 4 × 4 matrix can be decoupled to two 2 × 2 Hamiltonian matrices as shown in Eq. (1).

### Full-wave eigenmode analysis

The full-wave electrodynamics calculations were done with a FEM solver COMSOL Multiphysics 4.3a^27^. The eigenmode solver was employed to analyze the coupled propagation modes in the waveguide array. The medium used in this study are silica with a refractive index of 1.5 and silicon with a refractive index of 3.5.

## Additional Information

**How to cite this article**: Liu, Z. *et al.* Absence of Exceptional Points in Square Waveguide Arrays with Apparently Balanced Gain and Loss. *Sci. Rep.* **6**, 22711; doi: 10.1038/srep22711 (2016).

## Supplementary Material

Supplementary Information

## Figures and Tables

**Table 1:** The refractive index of the structure and the effective index for each waveguide.

*n*_0_	*n*_*A,B*_	*n*_*e*_	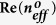	
Background medium	Waveguide medium	Fundamental mode of isolated waveguide	Transverse coupling case	Longitudinal coupling case
1.5	3.5	2.477	2.4763	2.4864
