# Supplementary material for: Absence of Exceptional Points in Square Waveguide Arrays with Apparently Balanced Gain and Loss
Source: Sci Rep. 2016 Mar 7;6:22711. doi: 10.1038/srep22711 (PMC4780277; doi:10.1038/srep22711)
Supplement: Supplementary Information [file srep22711-s1.pdf]

Electronic Supplementary Information

Absence of Exceptional Points in Square Waveguide Arrays with Apparently Balanced Gain and Loss

Zhenzhen Liu,[1] Qiang Zhang,[1] Xiangli Liu,[2] Y. Yao,[1] Jun-Jun Xiao[1,*]

[1]*College of Electronic and Information Engineering, Shenzhen Graduate School, Harbin Institute of Technology, Xili, Shenzhen 518055, China*

[2]*College of Materials Science and Engineering, Shenzhen Graduate School, Harbin Institute of Technology, Xili, Shenzhen 518055, China*

[*]Corresponding author: eiexiao@hitsz.edu.cn, xiangliliu@hit.edu.cn

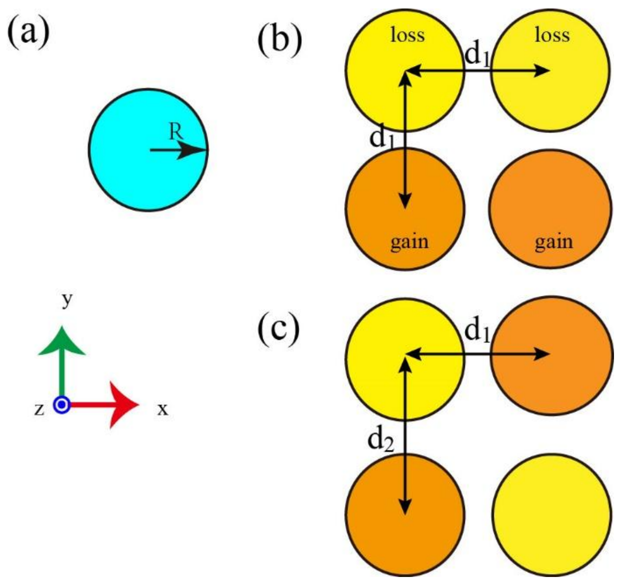

Figure S1. (a) A single circular waveguide and its coupling with the other ones in different cases: (b) with the distance $d_1 = d_2 = 0.5$ μm and gain/loss injected in parallel lines, (c) with the distance $d_1 = 0.5$ μm, $d_2 = 0.6$ μm and gain/loss injected diagonal.

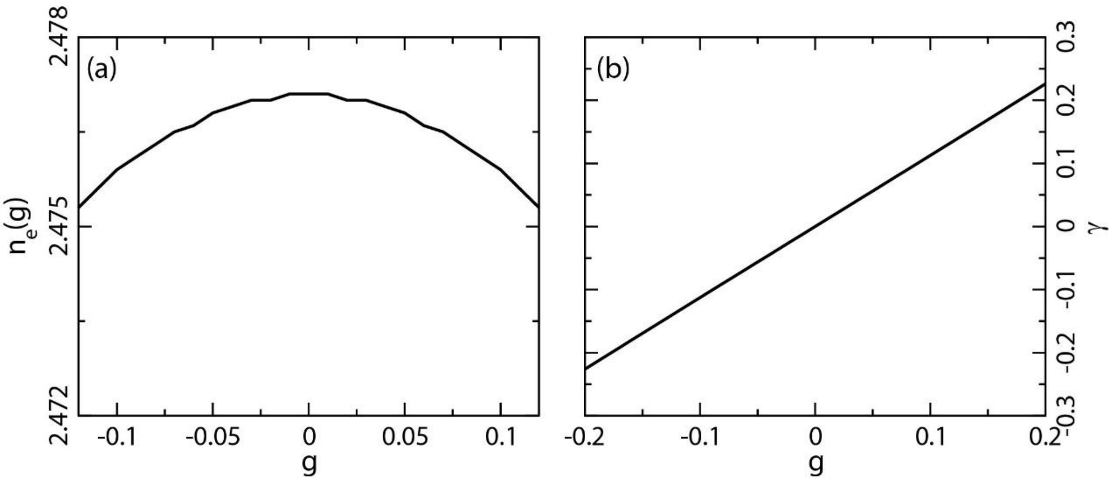

Figure S2. The real and imaginary part the effective index for a single waveguide (see Fig. S1a) as a function of gain/loss g**.** (a) The factor g has a small impact on the index of real part n_e. (b) The imaginary part of effective index γ is approximately in linear relation to g: $\gamma \approx 1.129g$.

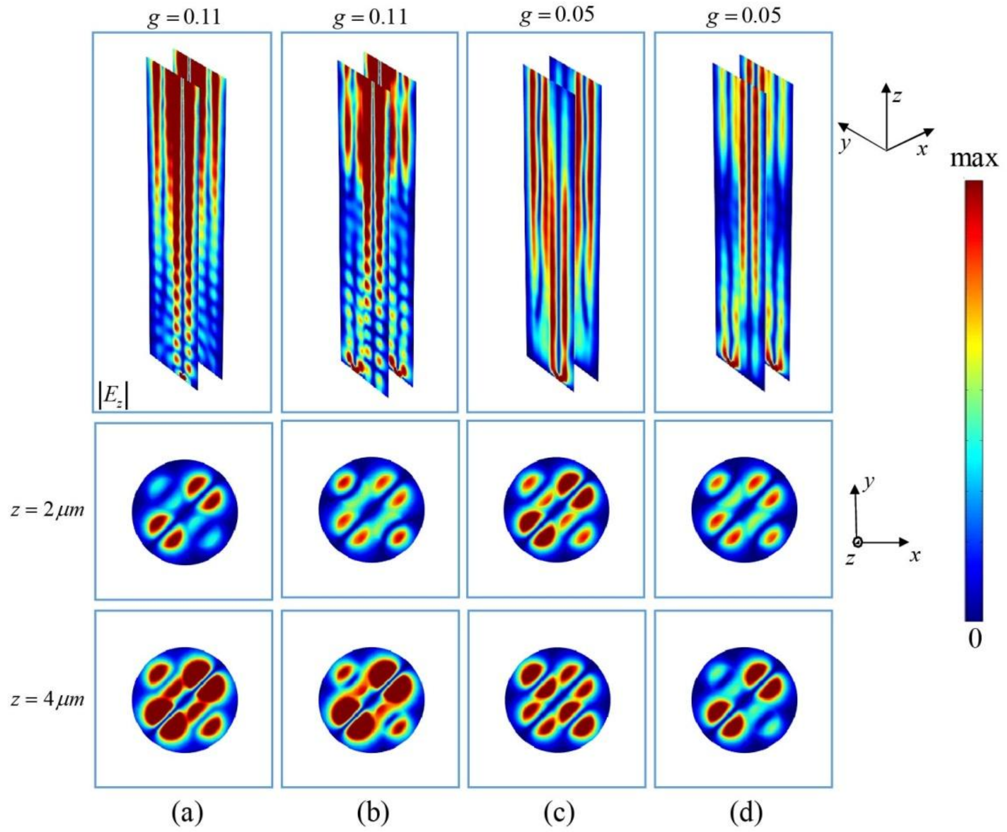

Figure S3. Simulated field intensity in the propagation direction (with a total length of 6 μm) and over the cross section planes at $z = 2$ μm and $z = 4$ μm for the system shown in Fig. 1d. Note that the input signal is launched selectively at the gain waveguides (a, c) and at the loss waveguides (b, d).

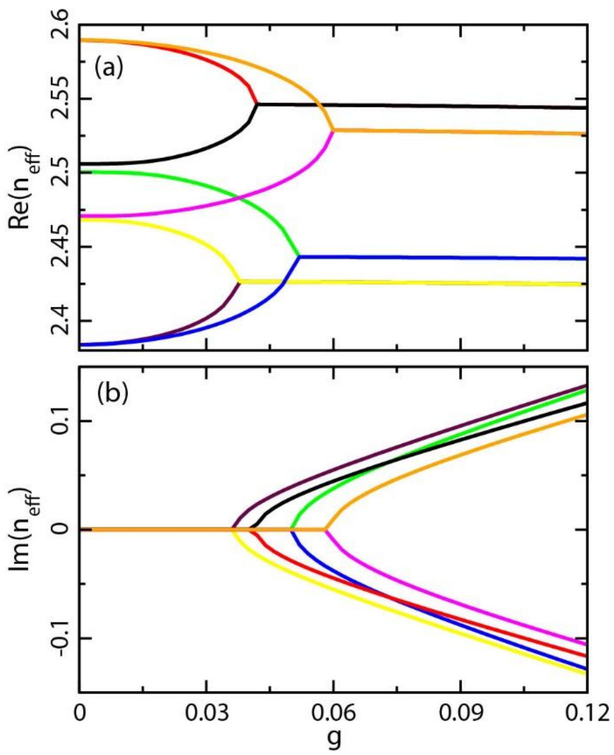

Figure S4. Evolution of eigenmode index trajectory as a function of gain/loss g for the configuration of Fig. S1b. (a) Real and (b) imaginary part of n_{eff} for the 8 guiding supermodes as a function of g. The results are obtained by FEM numerical simulation.

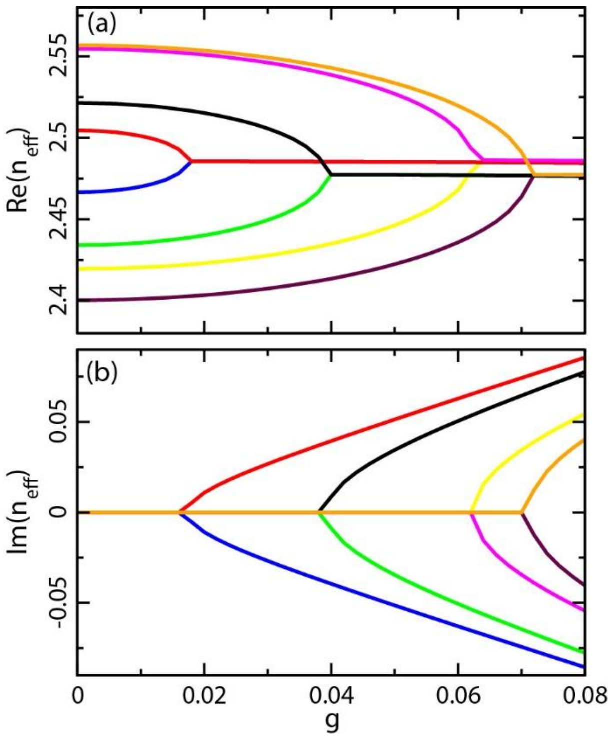

Figure S5. Similar to Figure S3, but for the configuration shown in Fig. S1c**.**
